# A General Model of Dioxin Contamination in Breast Milk: Results from a Study on 94 Women from the Caserta and Naples Areas in Italy

**DOI:** 10.3390/ijerph10115953

**Published:** 2013-11-08

**Authors:** Gaetano Rivezzi, Prisco Piscitelli, Giampiero Scortichini, Armando Giovannini, Gianfranco Diletti, Giacomo Migliorati, Roberta Ceci, Giulia Rivezzi, Lorenzo Cirasino, Pietro Carideo, Dennis M. Black, Carmine Garzillo, Umberto Giani

**Affiliations:** 1 St. Anna and St. Sebastiano Hospital, Via F. Palasciano, 81100 Caserta, Italy; E-Mail: pcaride@tin.it; 2 ISDE Campania, International Society Doctors for the Environment, 80100 Naples, Italy; E-Mail: priscofreedom@hotmail.com; 3 ISBEM, Euro Mediterranean Bio-Medical Scientific Institute, 72023 Brindisi, Italy; E-Mails: giulia.rivezzi@gmail.com (G.R.); cirasino@telnetwork.it (L.C.); 4 IZSAM, National Reference Laboratory for Dioxins and PCBs in Food and Feed, 64100 Teramo, Italy; E-Mails: g.scortichini@izs.it (G.S.); a.giovannini@izs.it (A.G.); g.diletti@izs.it (G.D.); g.migliorati@izs.it (G.M.); r.ceci@izs.it (R.C.); 5 Department of Epidemiology and Biostatistics, University of California, 185 Berry Street, San Francisco, CA 94107, USA; E-Mail: dblack@psg.ucsf.edu; 6 Department of Public Health, University Federico II, Via Pansini 5, 80131 Naples, Italy; E-Mails: carmine.garzillo@unina.it (C.G.); ugiani@unina.it (U.G.)

**Keywords:** dioxins, dioxin-like polychlorobiphenyls, persistent organic pollutants, human exposure, breastfeeding, environment, waste emergency

## Abstract

*Background*: The Caserta and Naples areas in Campania Region experience heavy environmental contamination due to illegal waste disposal and burns, thus representing a valuable setting to develop a general model of human contamination with dioxins (PCDDs-PCDFs) and dioxin-like-PCBs (dl-PCBs). *Methods*: 94 breastfeeding women (aged 19–32 years; mean age 27.9 ± 3.0) were recruited to determine concentrations of PCDDs-PCDFs and dl-PCBs in their milk. Individual milk samples were collected and analyzed according to standard international procedures. A generalized linear model was used to test potential predictors of pollutant concentration in breast milk: age, exposure to waste fires, cigarette smoking, diet, and residence in high/low risk area (defined at high/low environmental pressure by a specific 2007 WHO report). A Structural Equation Model (SEM) analysis was carried out by taking into account PCDDs-PCDFs and dl-PCBs as endogenous variables and age, waste fires, risk area and smoking as exogenous variables. *Results*: All milk samples were contaminated by PCDDs-PCDFs (8.6 pg WHO-TEQ/98g fat ± 2.7; range 3.8–19) and dl-PCBs (8.0 pg WHO-TEQ/98g fat ± 3.7; range 2.5–24), with their concentrations being associated with age and exposure to waste fires (*p* < 0.01). Exposure to fires resulted in larger increases of dioxins concentrations in people living in low risk areas than those from high risk areas (*p* < 0.01). *Conclusions*: A diffuse human exposure to persistent organic pollutants was observed in the Caserta and Naples areas. Dioxins concentration in women living in areas classified at low environmental pressure in 2007 WHO report was significantly influenced by exposure to burns.

## 1. Introduction

Dioxins include 210 congeners, mainly toxic compounds, recognized as environmental pollutants (Persistent Organic Pollutants, POPs) and forbidden since 2001 by the Stockholm Convention [1]. These molecules consist in polychlorinated dibenzo-*p*-dioxins (PCDDs) and polychlorinated dibenzofurans (PCDFs), hereafter referred to as dioxins. Polychlorinated biphenyls (PCBs) are chemically different from dioxins and include overall 209 congeners, 12 of which are dioxin-like PCBs (dl-PCBs) because they have “dioxin-like” properties [1]. Dioxins are not intentionally produced and mainly originated during burning processes such as waste incineration, forest fires, and industrial procedures and to a lesser extent as by-products of the production of pesticides. At the opposite, PCBs were globally manufactured and used in the past as components of paints, plasticizers, insulating materials, dielectric fluids and others until 1985 (when their use was forbidden). These compounds are widely diffused in the environment and can be found even far from the location where they are produced [1]. The most toxic chemical compound belonging to these classes of molecules is 2,3,7,8-tetrachlorodibenzo-*para*-dioxin (TCDD) [1]. Because dioxins and PCBs constitute a broad group of organochlorinated compounds that vary widely in toxicity, the concept of toxic equivalence (TEQ) has been developed to facilitate risk assessment and regulatory control. Toxic equivalence factors (TEFs) exist for selected congeners of dioxins and PCBs. Conventionally, the reference congener is the most toxic dioxin 2,3,7,8-TCDD, which has been assumed as having TEF = 1 [2,3]. In the assessment of dioxins and PCBs toxicity, different compounds are generally evaluated through toxicological equivalence. The highest environmental concentrations of dioxins are usually found in soil and sediment, while much lower levels are detectable in air and water [4,5]. Dioxins are lipid-soluble and accumulate in the fatty tissues (“bioaccumulation”), where they may persist for months or years [1].

Bioaccumulation is defined as a bioconcentration process, which can result in potentially toxic levels of these molecules even for human health. Humans are primarily exposed to dioxins and PCBs by eating food contaminated by these chemicals. Toxicological data indicate that about 80%–90% of human exposure is determined by contaminated food [1]. The most potent dioxin—2,3,7,8-TCDD has been classified as “a known human carcinogen” in the IARC (International Agency for Cancer Research) classification [6] since 1997. More recently, also 2,3,4,7,8-pentachlorodibenzofuran (2,3,4,7,8-PeCDF) and 3,3′,4,4′,5-pentachlorobiphenyl (PCB 126) have been recognized as carcinogenic substances [7].

The U.S. National Toxicology Program has stated that there is no known “safe dose” or “threshold” below which dioxins would not cause cancer [8], and a study published in July 2002 showed dioxins to be associated with an increased incidence of breast cancer [9]. Carcinogenic effects of dioxins have been described and documented at high dose exposures, such as after the Seveso accident [9,10,11,12] or during the exposure to the Agent Orange used in Vietnam War, although with mixed and inconsistent results among the different publications [13,14,15,16]. Long term exposure to lower dosages may also result in teratogenic effects, and can cause endocrine disruption, damage and suppression of the immune system, and severe reproductive and developmental problems, even at levels which are 100 times lower than those associated with carcinogenic effects [17,18,19,20,21,22,23,24]. *In utero* and lactational exposure of children to relatively low dosages of dioxins can permanently reduce sperm quality [25]. All PCBs have been classified as carcinogenic to humans (Group 1) [26].

A relevant source of dioxins emission in the environment (as important as other sources such as food and water) is represented by waste incineration and, owing to this reason, the regulatory authorities have fixed strict limitations to dioxins release in the air by waste incinerators (0.1 ng TEQ/Nm^3^), which does not actually correspond to the absence of risks for human health [27,28]. Illegal incineration of dangerous wastes and compounds containing chlorinated hydrocarbons (*i.e.*, tires and plastic materials) further contributes to enhance the exposure to dioxins and dioxin-like compounds. 

This is particularly relevant in some areas of Southern Italy interested by a “waste emergency”, particularly the area of Naples and Caserta, where the number of waste dumping sites in Campania has been officially estimated to exceed 6,000, with 60% of them being illegal and frequently containing toxic substances [29]. Some data are available about people’s exposure to potentially dangerous pollutants in these areas, including a report performed in 2007 by WHO and Italian healthcare authorities [30], and other valuable researches [31,32,33]. In the same areas, during the years 2001–2003, local health authorities detected high levels of dioxins contamination in cow milk and dairy products as well as in ground and soil [34]. It was possible to confirm that contamination of milk originated from dioxins-containing animal foods that were produced locally [35].

A recent study carried out in 2004 showed an excess of mortality rates in Naples and Caserta with respect to the national figures for lung, pleural, larynx, bladder, liver, and brain tumors [30]. As there were no waste incineration plants in the Campania region until year 2009, a possible correlation between these findings and uncontrolled incineration of urban or industrial wastes has been proposed [29,36]. Concentration of dioxins and PCBs in human milk have been suggested as a reliable model for the assessment of people’s exposure [37]. Actually, in human milk we can find many lipid-soluble compounds contained in the mother’s fatty tissues, so that dioxins and PCB detected levels should be regarded as representative of their concentration in fatty tissues and blood [38]. Moreover, human milk sample collection in breastfeeding women represents a simple and non-invasive method for determining the exposure to environmental contaminants, and the high lipid content of human milk allows a more accurate measurement of dioxins and PCBs compared to serum samples. However, several factors can affect the concentration of dioxins and PCBs in human milk, e.g., the mother’s age which can be considered as a proxy of the duration of the exposure, the dietary habits, the presence of illegal incineration of wastes, the place of living and other factors.

The present paper is aimed to develop a general model of human contamination with dioxins and dioxin-like-PCBs by analyzing milk samples from young breastfeeding women living in the Caserta and Naples areas.

## 2. Experimental Section

### 2.1. Patient Enrollment and Sample Analysis

Among all women delivering at the Department of Maternity and Neonatal Medicine of St. Anna and St. Sebastian Hospital in Caserta during the period of study (from June 2007 to May 2008), 104 consecutive breastfeeding women were recruited, but only 94 of them met all the criteria defined by the World Health Organization (WHO) for studies aimed to detect dioxins and PCB levels in human milk [18,39]: (1) age lower than 32 years old, (2) breastfeeding only one baby, (3) no previous deliveries, (4) no HIV/AIDS infection, (5) living in the same location for 5 years or more. All women who agreed to enter signed a specific informed consent. Eligible women were found to live in 45 towns located in Caserta and Naples provinces (none of the women came from the city of Naples). 

Data were collected through a personal interview carried out for each patient by the principal investigator; all exposures represented specific items in the questionnaire. The following data were collected for each woman: age (date of birth of the mother registered at the time of breast milk collection); weight (“what was your usual weight before the pregnancy?”); height; body mass index (BMI); previous weight loss (“have you experienced a weight loss >10 kg in the last 5 years?”); education level (“what is your degree of education?” *i.e.*, high school diploma; degree; intermediate schools; elementary school); current and previous occupation (“What is your current job? Have you changed job in the last 5 years? What kind of job it was?”); history of parental illnesses (“Has your mother suffered from specific diseases?”); number of cigarettes smoked (“Do you smoke or did you smoke in the year before the pregnancy? How many cigarettes per day did you smoke in average?”), weight of the newborn; distance between home and waste dumping sites (“Is your house nearby a dumping site?”; it must be noticed that investigators were aware of the presence of legal and illegal dumping sites in the town where patients lived in, even though the patient did not know about that); type and frequency of foods consumed during a standard week; a standard nutritional diary was administered to all the included women, in order to investigate if the following foods were eaten “never in a standard week”, “0–2 times per week”, “3–4 times per week”, “5 or more times per week”, “every day”: fish, vegetables, white meat, red meat, milk, dairy products; fresh dairy products, and eggs; at the same time, investigators asked if during a standard week, at least 50% of foods consisted in local products. Concerning exposure to fires, the question was: “have you ever personally observed any fires of wastes nearby your house in the last 5 years?”; the patient had the possibility to answer as follows: (a) no, never; (b) yes, sporadically; (c) yes, frequently. The question was about current exposure and exposures occurred in the last 5 years. The time window of 5 years has been chosen because the dioxins persist in the fatty tissue for several years (at least 10 years; so that 5 years roughly correspond to the dioxins halving time).

Milk samples were collected between two and eight weeks after the delivery. Each woman was provided with accurate instructions concerning how to collect and temporarily store the samples, in order to collect a minimum volume of 50 mL of milk during or immediately after breastfeeding, within a 48 h period. Each individual sample was separately stored in glass bottles at a temperature of +4 °C for a maximum time of 72 h. Then, each sample was frozen at −20 °C. Finally, samples were sent to the National Reference Laboratory for dioxins and PCBs in food and feed (IZSAM “G. Caporale”) in Teramo, where all chemical analyses were carried out on individual samples. A temperature below 20 °C was maintained during all the phases of the process (including the transfer of the samples from the hospital to the IZSAM) until the analyses were carried out. The samples were thawed and their lipid content was determined before undergoing purification and separation of dioxins and PCBs. Tests were carried out to search for PCDDs, PCDFs, and dl-PCB congeners. Toxic equivalent (WHO-TEQ98 for PCDDs-PCDFs and dl-PCBs) upper bound values were calculated using the toxic equivalent factor model proposed by WHO in 1997 [2,40]. Researched POPs are reported in Table 1. 

**Table 1:** List of pollutants determined in breast milk samples.

Dioxins	Dioxin-like PCBs
Polychlorodibenzo-*p*-dioxins(PCDDs)	non-*ortho*-PCBs
2,3,7,8-TCDD	TeCB-77
1,2,3,7,8-PeCDD	TeCB-81
1,2,3,4,7,8-HxCDD	PeCB-126
1,2,3,6,7,8-HxCDD	HxCB-169
1,2,3,7,8,9-HxCDD	
1,2,3,4,6,7,8-HpCDD	
OCDD	
Polychlorodibenzofurans (PCDFs)	mono-*ortho*-PCBs
2,3,7,8-TCDF	PeCB-105
1,2,3,7,8-PeCDF	PeCB-114
2,3,4,7,8-PeCDF	PeCB-118
1,2,3,4,7,8-HxCDF	PeCB-123
1,2,3,6,7,8-HxCDF	HxCB-156
2,3,4,6,7,8-HxCDF	HxCB-157
1,2,3,7,8,9-HxCDF	HxCB-167
1,2,3,4,6,7,8-HpCDF	HpCB-189
1,2,3,4,7,8,9-HpCDF	
OCDF	

### 2.2. Study Groups Characterization

**Figure 1:**
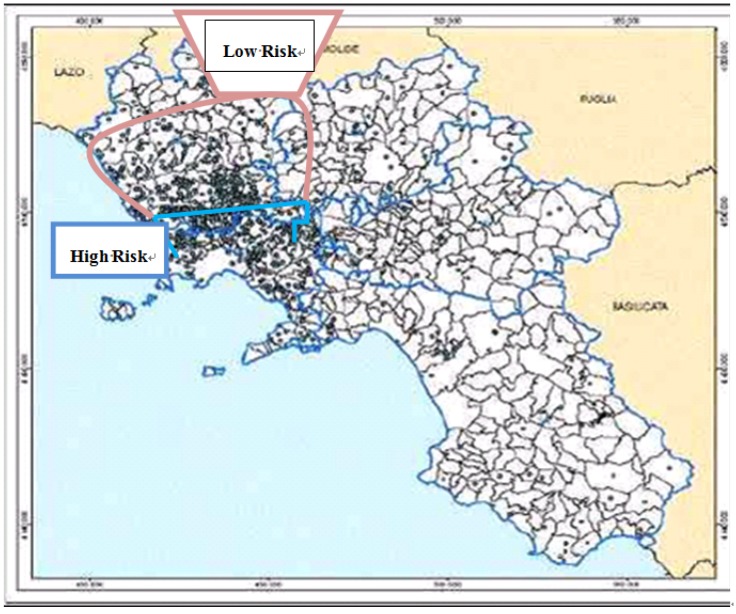
Characterization of study groups population. The borders of high-risk area and low-risk area are displayed on the map in blue and in red, respectively. The black points represent the dumping sites contaminated by toxic compounds according to the 2008 official report of Campania Regional Agency for the Environment (ARPAC).

A specific study (further referred as WHO_R) carried out in 2007 by WHO and several Italian institutions on behalf of the Civil Protection Department [41,42] categorized each town of Campania on a scale of 1 to 5, with class 5 corresponding to the highest environmental pressure, and excess of mortality for cancer. The geographic area between Naples and Caserta provinces was split into a high-risk area (WHO_R grade greater than 3) and a low-risk area (WHO_R grade from 1 to 3). Also, the high-risk zone corresponded to an area with a significant contamination by dioxins in ground and grass samples [34], cow/sheep/buffalo milk, animal feeds and dairy products [31,35] (Figure 1). Our study groups consisted in 44 women living in the high-risk area, and 50 women living in the low-risk area. 

### 2.3. Statistical Analyses

The sample size was stated before data gathering and was computed according to Cohen [43]. The sample size for the difference of the means of the dioxin’s concentrations were computed by setting α = 0.05, power = 0.8, effect size equal to 
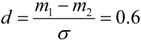
, *i.e.*, a medium effect size.

The required sample size was equal to 40 for each group. The sample size for the differences between the proportions of local food consumption in high risk area (P_1_) and the low risk area (P_2_) was oriented to detect a difference of 20% (*i.e.*, 0.3 *vs.* 0.1). The effect size was computed by transforming each proportion as:

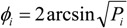

The desired effect size was computed as *h* = *ϕ*_1_ − *ϕ*_2_ = 0.6, *i.e.*, a medium effect size. By setting α = 0.05 and power = 0.8, the required sample size was equal to 44 for each group.

The normal distribution of dioxins and dl-PCBs was evaluated by means of the Kolmogorov-Smirnov test. The correlations between age and dioxins, age and dl-PCBs, age and fat content in the maternal milk, were evaluated by means of the Pearson correlation coefficient. 

Pearson’s χ^2^ was used to study the independence of the source (mostly local or national) of food consumption with risk area and exposure to waste fires, respectively. A multivariate generalized linear model (GLM) was used to test potential predictors of pollutant concentrations in breast milk. In order to avoid combinations of independent variables with insufficient number of cases, two different GLM analyses were carried out. The first one aimed to test whether the source of foods (fish, vegetables, milk, cheese and dairy products, meat) affected the concentration of pollutants in the maternal milk. Local egg consumption *vs.* risk area was not taken into account due to the insufficient number of cases in high risk areas. Since no statistical association was found between the source of foods and dioxins or dl-PCBs, only age and environmental potential factors, *i.e.*, exposure to waste fires (yes/no), living in high-risk or low-risk area and cigarette smoking (more than twice a day) were considered as predictors in the second analysis. In both cases the GLM was carried out by taking dioxins and dl-PCBs as dependent variables. The main effects and the second level of interactions were tested. The significance level was fixed at 0.05. 

In order to formulate a general model, the results of the GLM analysis were integrated with a Structural Equation Model (SEM) analysis [44] carried out by taking into account dioxins and dl-PCBs as endogenous variables and age, waste fires, risk area and smoking as exogenous variables. Unlike GLM, SEM analysis supplies information as a causal modeling of the processes under study, represented by a series of structural (*i.e.*, regression) equations, which can be also modeled pictorially to enable a clearer conceptualization of the theory. Moreover, unlike most other multivariate procedures that are essentially descriptive by nature, it takes a confirmatory rather than an exploratory approach to the data analysis. Whereas traditional multivariate procedures are incapable of either assessing or correcting for measurement error, SEM provides explicit estimates of these error variance parameters. The IBM_SPSS_20 and AMOS software packages were used for the statistical analyses.

## 3. Results and Discussion

### 3.1. Results

The mean age was 27.9 ± 3.0 years (range 19–32). The overall average concentration of dioxins in mother’s milk was 8.6 pg WHO-TEQ98/g fat ± 2.7 (range 3.8–19.0), with 2,3,7,8-TCDD levels being 0.77 pg WHO-TEQ98/g fat ± 0.30 (range 0.30–2.2). The average dl-PCB concentration was 8.0 pg WHO-TEQ98/g fat ± 3.7 (range 2.5–24). Table 2 summarizes main characteristics of women enrolled in the study, including average concentration of pollutants found in their milk. Both dioxins and dl-PCBs were normally distributed (respectively, K-S z = 0.76, *p* = 0.61, and K-S z = 1.02, *p* = 0.24). 

**Table 2:** Characteristics of enrolled women, including average concentration of pollutants found in their milk samples. (N=Number of cases; Information is not available for 1 subject).

Parameter	Value	N	Dioxins (pg WHO-TEQ98/g fat) (mean ± S.D.)	dl-PCBs (pg WHO-TEQ98/g fat) (mean ± S.D.)
Age (years)	19–24	12	6.40 ± 2.98	5.20 ± 2.45
	25–29	49	8.43 ± 2.43	8.29 ± 3.80
	30–32	33	9.53 ± 2.01	8.69 ± 2.45
Smoking	Yes	25	7.70 ± 2.50	6.70 ± 3.00
	No	68	8.87 ± 2.72	8.51 ± 3.79
Exposure to fires	Yes	13	10.5 ± 3.10	9.91 ± 3.39
	No	81	8.25 ± 2.52	7.73 ± 3.60
Area	High Risk	44	9.30 ± 2.76	8.05 ± 3.58
	Low Risk	50	7.91 ± 2.45	8.02 ± 3.64

Pearson correlations between age and dioxins (r = 0.437, *p* = 0.003) and age and dl-PCBs (r = 0.351, *p* = 0.001) were statistically significant. These significant correlations disappeared in the low-risk area if exposure to waste fires was present. Table 3 shows the independence of the majority of food consumption with risk area and waste fire exposure. Only one woman living in high-risk area declared local egg consumption.

No significant associations between dioxins or dl-PCBs concentrations in breast milk and source of foods were found (Table 4).

**Table 3:** Pearson’s χ^2^ test on source of food consumption *vs.* exposure to waste fires and *vs.* risk zone. NA = not applicable.

	Local (yes/no)	Pearson χ^2^ Test			
		Exposure to Fires	*p*	Area (high/low risk)	*p*
Fish	23/67	0.217	0.733	0.017	0.897
Vegetables	70/23	0.296	0.729	1.323	0.336
Milk	15/73	0.011	0.915	3.215	0.092
Cheese	33/58	0.759	0.525	0.484	0.486
Meat	52/42	0.513	0.554	0.476	0.537
Eggs	20/73	0.335	0.562	== ^1^	NA

**^1^** Not considered because only one woman living in high-risk area declared local egg consumption.

**Table 4:** Generalized linear model. Tests of between-subjects effects on source of food consumption like: fish, vegetables, milk, cheese, meat, and eggs.

Source	Dependent Variable	F Ratio	*p*
Fish	Dioxins	0.006	0.936
	dl-PCBs	0.677	0.415
Vegetables	Dioxins	0.460	0.501
	dl-PCBs	0.661	0.420
Milk	Dioxins	0.018	0.893
	dl-PCBs	0.433	0.514
Cheese	Dioxins	0.187	0.667
	dl-PCBs	0.002	0.965
Meat	Dioxins	0.570	0.454
	dl-PCBs	1.167	0.285
Eggs	Dioxins	0.944	0.336
	dl-PCBs	0.035	0.853
Fish * Fires	Dioxins	3.650	0.060
	dl-PCBs	2.005	0.161
Fish * Area	Dioxins	2.578	0.113
	dl-PCBs	3.425	0.069
Vegetables * Fires	Dioxins	0.245	0.622
	dl-PCBs	0.055	0.814
Vegetables * Area	Dioxins	1.529	0.221
	dl-PCBs	1.216	0.274
Milk * Fires	Dioxins	2.916	0.092
	dl-PCBs	0.558	0.458
Milk * Area	Dioxins	0.048	0.827
	dl-PCBs	1.076	0.303
Cheese * Fires	Dioxins	0.350	0.556
	dl-PCBs	1.518	0.222
Cheese * Area	Dioxins	0.002	0.960
	dl-PCBs	0.949	0.334
Meat * Fires	Dioxins	2.899	0.093
	dl-PCBs	0.276	0.601
Meat * Area	Dioxins	0.916	0.342
	dl-PCBs	0.026	0.872

**Figure 2:**
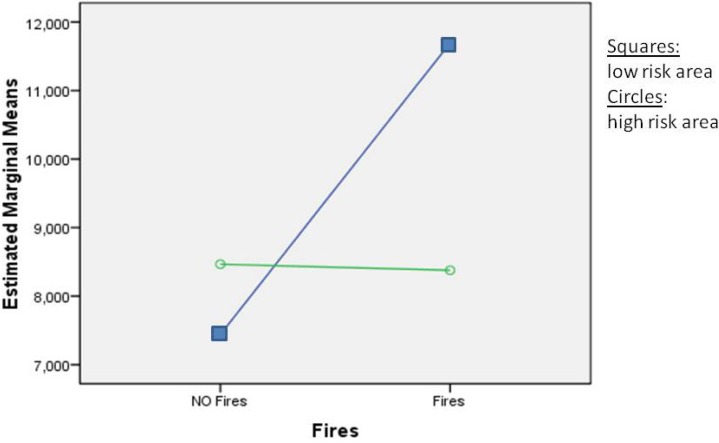
Dioxins Estimated Marginal Means (pg WHO-TEQ98/g of fat). To be noticed thatcovariates presented in the model have been evaluated at the following values: Age = 27.9 years.

Table 5 shows that age and exposure to waste fires were statistically associated to dioxins. Also, there was a significant interaction between exposure to waste disposal fires and risk area, such that in the low-risk area the dioxins level was higher in presence of exposure to fires, while this effect was not detected in the high-risk area. In low-risk area milk contamination was more related to individual exposure to fires (Figure 2), thus suggesting that in high-risk area the effect of fire exposure is combined with many other factors that produce a diffuse basal level of dioxins contamination in the environment, while in low-risk areas the contribution of personal exposure to fires in determining milk contamination is more clearly distinguished. This also indicates that the WHO_R risk classification of environmental pressure in Campania Region towns did not taken into account the burden of illegal waste fires. Concentrations of dioxin-like PCBs were only associated to age.

**Table 5:** Generalized linear model. Between-Subjects Effects on environmental and age variables.

Source	Dependent Variable	F Ratio	*p*
Age	Dioxins	20.005	0.000
	dl-PCBs	7.214	0.009
Smoking	Dioxins	0.943	0.334
	dl-PCBs	2.401	0.125
Exposure to Fires	Dioxins	8.423	0.005
	dl-PCBs	3.788	0.055
Area (high/low risk)	Dioxins	2.834	0.096
	dl-PCBs	1.343	0.250
Smoking * Fires	Dioxins	1.686	0.198
	dl-PCBs	0.029	0.865
Smoking * Age	Dioxins	1.767	0.099
	dl-PCBs	0.494	0.857
Age * Fires	Dioxins	1.264	0.296
	dl-PCBs	0.398	0.809
Age * Area	Dioxins	1.813	0.090
	dl-PCBs	0.591	0.782
Smoking * Area	Dioxins	0.284	0.596
	dl-PCBs	0.337	0.564
Fires * Area	Dioxins	11.763	0.001
	dl-PCBs	2.888	0.093

**Figure 3:**
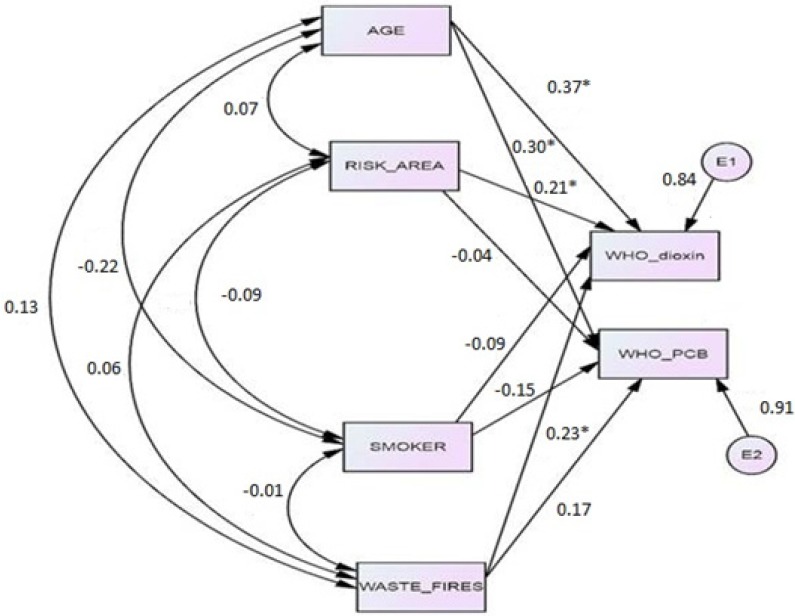
Path Diagram (Significant Standardized Regression Weights are asterisked).

The path coefficients of the SEM model show that dioxins and dl-PCBs in maternal milk have different determinants (Table 6 and Figure 3). The dioxins concentration is significantly affected by age (path coefficient = 0.37), waste fires (path coefficient = 0.23), risk area (path coefficient = 0.21). Dioxin-like PCBs were affected only by age (path coefficient = 0.30).

**Table 6:** Standardized Regression Weights Estimations, presented with their *p*-values as appearing in Path Diagram.

Determinant			Standardized Regression Weights	*p*
WHO-TEQ98-dl-PCBs	<---	Age	0.298	0.002
WHO-TEQ98-dioxins	<---	Age	0.371	0.001
WHO-TEQ98-dioxins	<---	Risk_area	0.213	0.015
WHO-TEQ98-dl-PCBs	<---	Risk_area	−0.041	0.665
WHO-TEQ98-dl-PCBs	<---	Smoker	−0.154	0.113
WHO-TEQ98-dioxins	<---	Smoker	−0.089	0.320
WHO-TEQ98-dioxins	<---	Waste_fires	0.226	0.010
WHO-TEQ98-dl-PCBs	<---	Waste_fires	0.168	0.079

### 3.2. Discussion

This is the largest study carried out in Italy on breastfeeding women to detect dioxins and dl-PCBs contamination. This is also one of the few studies where only individual human milk samples have been examined to get specific information about individual risk factors. An added value—for public health purposes—is that this study has analyzed women living in Naples and Caserta areas, which have undergone heavy environmental pollution in the last decades, as documented by official regional [34], national [27,30,34,45], and NATO [31] surveys. 

Our findings were comparable to dioxins and dl-PCBs contamination observed in Sweden [46]. Contaminated municipalities from the Caserta and Naples areas in our study showed higher dioxins and dl-PCBs concentrations (upper bound values) than those observed in Milan, Piacenza, and Giugliano (a town nearby Naples) by Ulaszewska *et al.* [47], and levels measured in China [38,48], Norway [49], Greece [50], Bavaria [51] and Australia (all pooled samples; [52]). Particular attention should be paid to the results from Ulaszewska *et al.* [47], which included the town of Giugliano in Campania, taking into account that dioxins concentrations in that study were expressed in the new format of WHO toxicity equivalent factors (WHO-TEQ05-PCDDs-PCDFs). Dioxins contamination in breast milk found by Ulaszewska *et al.* [47] were in the same range in the three cities, but with higher mean values in Milan and Piacenza than in Giugliano (mean and range being the following: Milan: 4.70, 2.42–9.55; Piacenza: 4.67, 2.43–7.70; Giugliano: 3.78, 1.26–9.44). Our study included six mothers from Giugliano in Campania and their mean and range of contamination level, corresponded to 6.40 and 4.07–8.43 respectively (when expressed in the new format of WHO-TEQ05-PCDDs-PCDFs). Our study also included other municipalities of Caserta and Naples provinces. Overall, the contamination detected in our study had mean levels and range of 6.53 and 2.89–14.64 respectively, when expressed in the new format of WHO-TEQ05-PCDDs-PCDFs. 

This information clearly indicated that the average contamination of the study area of Caserta and Naples is similar to that of the cities of Milan and Piacenza, but the highly contaminated municipalities of Caserta and Naples have dioxins concentrations that are up to 1.5 times the maximum contamination recorded in Milan by Ulaszewska *et al.* [47]. However, we found a lower concentration of dioxins and dl-PCBs with respect to studies carried out in Duisburg (Germany) on individual samples [53]. Table 7 summarizes the findings of all the major studies carried out in different countries.

Dioxins and dl-PCBs levels in breast milk were directly correlated with the age of the enrolled women. There was no correlation between age and fat content in the maternal milk (Pearson coefficient = −0.079). Taking into account that the increase in colostrum fat content occurs in mothers with advanced age (≥35 years) [54], due to increased fat synthesis and excretion in milk, reduced water content of milk or a combination of both factors, the correlation between age and pollutant concentration could be considered an effect of the duration of exposure to these compounds, thus confirming their bioaccumulation properties. In other words, the age can be considered as a proxy of the length of the exposure. This point is of particular interest because all women were young (under 32 years old), and also because no legal waste incineration plants, large metallurgy plants or other industries known as sources of dioxins and PCBs were present in the examined area at the time of the study. A longer duration of exposure (since very young age) to a diffuse illegal street incineration practice of wastes, tires, plastics, or other toxic materials, and small industrial activities may all have played an important role in determining bioaccumulation of the substances under investigation. This hypothesis is supported by the significant association between exposure to waste fires and higher concentrations of dioxins in the milk samples. 

**Table 7:** Levels of PCDDs-PCDFs and dl-PCBs in breast milk in different countries.

Reference	Country/Study area	Dioxins (pg WHO-TEQ98/g fat)	dl-PCBs (pg WHO-TEQ98/g fat)	Dioxins (pg WHO-TEQ05/g fat)	dl-PCBs (pg WHO-TEQ05/g fat)
This study	Italy/Caserta and Naples	8.60	8.00		
Ulazewska *et al.*, 2011 [47]	Italy				
	Milan			4.70	6.28
	Piacenza			4.67	5.27
	Giugliano			3.78	4.87
Li *et al.*, 2009 [48]	China	3.73 *	1.70 *	3.12 *	1.47 *
Lignell *et al.*, 2009 [46]	Sweden	8.20		7.00	
Raab *et al.*, 2007 [51]	Germany/Bavaria	9.91	9.88		
Harden *et al.*, 2007 [52]	Australia	5.90	3.10		
Wittsiepe *et al.*, 2007 [53]	Germany/Duisburg	13.84	13.43		
Costopoulou *et al.*, 2006 [50]	Greece/Athens	7.27	3.48		
Hedley *et al.*, 2006 [38]	China/Hong Kong	8.25	4.67		
Becher *et al.*, 1995 [49]	Norway	10.40			
	Lithuania	10.80			

* Weighted mean values recalculated from original data related to different areas of study.

Table 5 shows that the main effect of the risk area was not statistically significant (respectively *p* = 0.096 for dioxins and *p* = 0.25 for dl-PCBs). Actually, dioxins concentration was affected by the risk area just when testing the interaction with exposure to waste fires. Figure 2 shows that dioxins concentration in the low-risk area was significantly higher in presence of illegal incineration of wastes. The significant interaction between area and fires indicates that fire exposure was a more important determinant of the milk concentration of dioxins in low-risk areas compared to high-risk areas. These effects have been quantified by the regression coefficients reported in Table 6 and in Figure 3. SEM analysis shows that dioxins concentration is directly influenced by the risk area. It is interesting to point out that age is the most crucial factor associated to higher concentration of both dioxins and dl-PCBs in maternal milk, but exposure to fires influences only dioxins concentration. This can be explained by the different mechanism of contamination of these two major pollutants. The role of consumption of cow/sheep/buffalo milk or dairy products was not statistically associated with dioxins and dl-PCBs. Having found no significant associations between dioxins or dl-PCBs concentrations in breast milk and type/frequency of food consumed is consistent with the uniformity of dietary habits in Campania. Actually, we did not expect to find differences in the frequency of different foods consumption between small towns of the same region. 

Finally, the possibility of dioxins and dl-PCBs intake by newborns through maternal milk should be considered as already suggested by a recently published paper of Bianco et al. [55]. Consistently with a study carried out in a German industrial area [53], the mean daily intake of milk contaminants (PCDDs, PCDFs, and dl-PCBs) by the newborns through maternal milk was in our study estimated at about 90.4 pg WHO-TEQ98/kg body weight/day, while the WHO maximum acceptable threshold is 20 pg for a 5 kg baby (1–4 pg WHO-TEQ98/kg body weight/day). 

## 4. Conclusions

A general model of the determinants of dioxins and dl-PCBs contamination of breast milk was assessed. The levels of contaminants detected in all individual samples suggest long-lasting diffuse human exposures in the Caserta and Naples area, which is still experiencing “waste emergency”. A specific effect attributable to local exposure to illegal waste burning and living in low-risk area (according to 2007 WHO Report, [41,42]), was clearly observed for dioxins, unlikely from dl-PCBs. Bioaccumulation of pollutants seems to have occurred in humans over less than three decades so that the age can be considered a proxy for the duration of the exposure to both the pollutants, independently from any dietary habit. It would be interesting to determine the burden the new waste incineration plant located in Acerra, and other plants under construction in the same area.

## References

[1] Communication from the Commission to the Council, the European Parliament and the Economic and Social Committee Community—Strategy for Dioxin, Furans, and Polychlorinated Biphenyls. http://eurlex.europa.eu/smartapi/cgi/sga_doc?smartapi!celexplus!prod!DocNumber&lg=en&type_doc=COMfinal&an_doc=2001&nu_doc=593.

[2] van den Berg M., Birnbaum L., Bosveld A.T.C., Brunstrom B., Cook P., Feely M., Giesy J.P., Hanberg A., Hasegawa R., Kennedy S.W. (1998). Toxic equivalency factors (TEFs) for PCBs, PCDDs, PCDFs for humans and wildlife. Environ. Health Perspect..

[3] van den Berg M., Birnbaum L., Denison M., de Vito M., Farland W., Feeley M., Fiedler H., Hakansson H., Hanberg A., Haws L. (2006). The 2005 World Health Organization reevaluation of human and mammalian toxic equivalency factors for dioxins and dioxin-like compounds. Toxicol. Sci..

[4] Fiedler H. (1996). Sources of PCDDIPCDF and impact on the environment. Chemosphere.

[5] Dyke P.H., Foanbj C., Wenborn M., Colemanc P.J. (1997). Review of dioxin releases to land and water in the UK. Sci. Total Environ..

[6] IARC (1997). Polychlorinated dibenzo-*para*-dioxins and polychlorinated dibenzofurans. IARC Monographs on the Evaluation of Carcinogenic Risks to Humans.

[7] IARC (2012). A review of human carcinogens—Part F: Chemical agents and related occupations. IARC Monographs on the Evaluation of Carcinogenic Risks to Humans.

[8] Steenland K., Bertazzi P., Baccarelli A., Kogevinas M. (2004). Dioxin Revisited: Developments since the 1997 IARC Classification of Dioxin as a human carcinogen. Environ. Health Perspect..

[9] Warner M., Eskenazi B., Mocarelli P., Gerthoux P.M., Samuels S., Needham L., Patterson D., Brambilla P. (2002). Serum dioxin concentrations and breast cancer risk in the Seveso women’s health study. Environ. Health Perspect..

[10] Consonni D., Pesatori A.C., Zocchetti C., Sindaco R., D’ORO L.C., Rubagotti M., Bertazzi P.A. (2008). Mortality in a population exposed to dioxin after the Seveso, Italy, accident in 1976: 25 years of follow-up. Amer. J. Epidemiol..

[11] Pesatori A.C., Consonni D., Rubagotti M., Grillo P., Bertazzi P.A. (2009). Cancer incidence in the population exposed to dioxin after the “Seveso accident”: Twenty years of follow-up. Environ. Health.

[12] Warner M., Mocarelli P., Samuels S., Needham L., Brambilla P., Eskenazi B. (2011). Dioxin exposure and cancer risk in the Seveso women’s health study. Environ. Health Perspect..

[13] Ketchum N.S., Michalek J.E., Burton J.E. (1999). Serum dioxin and cancer in veterans of operation ranch hand. Amer. J. Epidemiol..

[14] Pavuk M., Michalek J.E., Schecter A., Ketchum N.S., Akhtar F.Z., Fox K.A. (2005). Did TCDD exposure or service in Southeast Asia increase the risk of cancer in air force Vietnam veterans who did not spray agent orange?. J. Occup. Environ. Med..

[15] Michalek J.E., Pavuk M.J. (2008). Diabetes and cancer in veterans of operation ranch hand after adjustment for calendar period, days of spraying, and time spent in Southeast Asia. Occup. Environ. Med..

[16] Ansbaugh N., Shannon J., Mori M., Farris P.E., Garzotto M. (2013). Agent Orange as a risk factor for high-grade prostate cancer. Cancer.

[17] Irigaray P., Newby J.A., Clapp R., Hardell L., Howard V., Montagnier L., Epstein S., Belpomme D. (2007). Lifestyle-related factors and environmental agents causing cancer: An overview. Biomed. Pharmacotherapy.

[18] Lei Z., Rui Z., Qing S.Y., Ling Z., Ning W.Y., Yi Z. (2013). Synergistic effects of 2,3,7,8-tetrachlorodibenzo-p-dioxin and N-nitrosodiethylamine on cell malignant transformation. Biomed. Environ. Sci..

[19] Lundqvist C., Zuurbier M., Leijs M., Johansson C., Ceccatelli S., Saunders M., Schoeters G., Tusscher G.T., Koppe J.G. (2006). The effects of PCBs and dioxins on child health. Acta. Paediat..

[20] Viel J.F., Floret N., Deconinck E., Focant J.F., de Pauw E., Cahn J.Y. (2011). Increased risk of non-Hodgkin lymphoma and serum organochlorine concentrations among neighbors of a municipal solid waste incinerator. Environ. Int..

[21] Wang L., Weng S., Wen S., Shi T., Sun G., Zeng Y., Qi C., Chen W. (2013). Polychlorinated dibenzo-p-dioxins and dibenzofurans and their association with cancer mortality among workers in one automobile foundry factory. Sci. Total Environ..

[22] Fletcher C., Unni K.K., Mertens F. (2002). Pathology and Genetics of Tumours of Soft Tissue and Bone.

[23] Leeuwen F.X.R.V., Malisch R. (2002). Results of the third round of the WHO coordinated exposure study on the levels of PCBs, PCDDs, and PCFDs in human milk. Organohalogen Compd..

[24] Scientific Committee on Food (SCF) (2000). Opinion of the SCF on the risk assessment of dioxins and dioxin-like PCBs. http://ec.europa.eu/food/fs/sc/scf/out78_en.pdf.

[25] Mocarelli P., Gerthoux P.M., Needham L.L., Patterson D.G., Limonta G., Falbo R., Signorini S., Bertona M., Crespi C., Sarto C. (2011). Perinatal exposure to low doses of dioxin can permanently impair human semen quality. Environ. Health Perspect..

[26] Lauby-Secretan B., Loomis D., Grosse Y., El Ghissassi F., Bouvard V., Benbrahim-Tallaa L., Guha N., Baan R., Mattock H., Straif K. (2013). Carcinogenicity of polychlorinated biphenyls and polybrominated biphenyls. Lancet Oncol..

[27] Population Health and Waste Management: Scientific Data and Policy Options. http://www.euro.who.int/__data/assets/pdf_file/0012/91101/E91021.pdf.

[28] Directive 2000/76/EC of the European Parliament and of the Council of 4 December 2000 on the Incineration of Waste. http://eur-lex.europa.eu/LexUriServ/LexUriServ.do?uri=OJ:L:2000:332:0091:0091:EN:PDF.

[29] Annuario dei Dati Ambientali in Campania 2006. http://www.arpacampania.it/documents/30626/51722/Siti+Contaminati.pdf.

[30] Fazzo L., Belli S., Minichilli F., Mitis F., Santoro M., Martina L., Pizzuti R., Comba P., Martuzzi M., Bianchi F. (2008). Cluster analysis of mortality and malformations in the provinces of Naples and Caserta (Campania Region). Ann. Ist. Super. Sanità.

[31] Diletti G., Ceci R., Conte A., de Benedictis A., Migliorati G., Scortichini G., Mehmetli E., Koumanova B. (2008). Milk contamination from dioxins in Italy: Source identification and intervention strategies. Fate of Persistent Organic Pollutants in the Environment.

[32] Esposito M., Serpe F.P., Neugebauer F., Cavallo S., Gallo P., Colarusso G., Baldi L., Iovane G., Serpe L. (2010). Contamination levels and congener distribution of PCDDs, PCDFs and dioxin-like PCBs in buffalo’s milk from Caserta province (Italy). Chemosphere.

[33] Esposito M., Cavallo S., Serpe F.P., D’Ambrosio R., Gallo P., Colarusso G., Pellicanò R., Baldi L., Guarino A., Serpe L. (2009). Levels and congener profiles of polychlorinated dibenzo-p-dioxins, polychlorinated dibenzofurans and dioxin-like polychlorinated biphenyls in cow’s milk collected in Campania, Italy. Chemosphere.

[34] Concentrazione Totale di Diossina e Furani Rilevati sulla Matrice Terreno. http://www.arpacampania.it/documents/30626/56429/Diossina_A4.pdf.

[35] Diletti G., Torreti L., de Massis M.R., Migliorati G., Scortichini G. (2003). A case of milk contamination by PCDD/Fs in Italy: Analytical levels and contamination source identification. Organohalogen Compd..

[36] Bianchi F., Comba P., Martuzzi M., Palombino R., Pizzuti R. (2004). Reflection and Reaction: Italian “Triangle of death”. Lancet Oncol..

[37] LaKind J.S., Berlin C.M., Naiman D.Q. (2001). Infant exposure to chemicals in breast milk in the United States: What we need to learn from a breast milk monitoring program. Environ. Health Perspect..

[38] Hedley A.J., Wong T.W., Hui L.L., Malisch R., Nelson E.A. (2006). Breast Milk Dioxins in Hong Kong and Pearl River Delta. Environ. Health Perspect..

[39] Fourth WHO-Coordinated Survey of Human Milk for Persistent Organic Pollutants in Cooperation with UNEP: Guidelines for Developing a National Protocol. http://www.who.int/foodsafety/chem/POPprotocol.pdf.

[40] Polychlorinated Dibenzoparadioxins and Polychlorinated Dibenzofurans. http://apps.who.int/bookorders/anglais/detart1.jsp?sesslan=1&codlan=1&codcol=72&codcch=69#(accessed.

[41] Trattamento dei Rifiuti in Campania: Impatto sulla Salute Umana. http://www.protezionecivile.gov.it/jcms/it/view_dossier.wp?contentId=DOS14955.

[42] Martuzzi M., Mitis F., Bianchi F., Minichilli F., Comba P., Fazzo L. (2009). Cancer mortality and congenital anomalies in a region of Italy with intense environmental pressure due to waste. Occup. Environ. Med..

[43] Cohen J. (1988). Statistical Power Analysis for Behavioral Sciences.

[44] Pugesek B.H., Tomer A., von Eye A. (2003). Structural Equation Modeling: Applications in Ecological and Evolutionary Biology.

[45] Diossine, Furani e Policlorobifenili: Indagine Ambientale nella Regione Campania. http://www.isprambiente.gov.it/files/pubblicazioni/quaderni/laboratorio/Quad_Lab_1_2012.pdf.

[46] Lignell S., Aune M., Darnerud P.O., Cnattingius S., Glynn A. (2009). Persistent organochlorine and organobromine compounds in mother’s milk from Sweden 1996–2006: Compound-specific temporal trends. Environ. Res..

[47] Ulaszewska M.M., Zuccato E., Capri E., Iovine R., Colombo A., Rotella G., Generoso C., Grassi P., Melis M., Fanelli R. (2011). The effect of waste combustion on the occurrence of polychlorinated dibenzo-p-dioxins (PCDDs), polychlorinated dibenzofurans (PCDFs) and polychlorinated biphenyls (PCBs) in breast milk in Italy. Chemosphere.

[48] Li J., Zhang L., Wu Y., Liu Y., Zhou P., Wen S., Liu J., Zhao Y., Li X. (2009). A national survey of polychlorinated dioxins, furans (PCDD/Fs) and dioxin-like polychlorinated biphenyls (dl-PCBs) in human milk in China. Chemosphere.

[49] Becher G., Skaare J.U., Polder A., Sletten B., Rossland O.J., Hansen H.K., Ptashekas J. (1995). PCDDs, PCDFs, and PCBs in human milk from different parts of Norway and Lithuania. J. Toxicol. Environ. Health.

[50] Costopoulou D., Vassiliadou I., Papadopoulous A., Makropoulos V., Leondiadis L. (2006). Levels of dioxins, furans and PCBs in human serum and milk of people living in Greece. Chemosphere.

[51] Raab U., Schwegler U., Preiss U., Albrecht M., Fromme H. (2007). Bavarian breast milk survey—Pilot study and future developments. Int. J. Hyg. Environ. Health.

[52] Harden F.A., Toms L.M.L., Symons R., Fürst P., Berry Y., Müller J.F. (2007). Evaluation of dioxin-like chemicals in pooled human milk samples collected in Australia. Chemosphere.

[53] Wittsiepe J., Furst P., Schrey P., Lemm F., Kraft M., Eberwein G., Winneke G., Wilhelm M. (2007). PCDD/F and dioxin-like PCB in human blood and milk from German mothers. Chemosphere.

[54] Kedem M.H., Mandel D., Domani K.A., Mimouni F.B., Shay V., Marom R., Dollberg S., Herman L., Lubetzky R. (2013). The effect of advanced maternal age upon human milk fat content. Breastfeeding Med..

[55] Bianco G., Zianni R., Anzillotta G., Palma A., Vitacco V., Scrano L., Cataldi T.R. (2013). Dibenzo-p-dioxins and dibenzofurans in human breast milk collected in the area of Taranto (Southern Italy): First case study. Anal. Bioanal. Chem..

